# May Salivary Chromogranin A Act as a Physiological Index of Stress in Transported Donkeys? A Pilot Study

**DOI:** 10.3390/ani10060972

**Published:** 2020-06-03

**Authors:** Francesca Dai, Emanuela Dalla Costa, Simona Cannas, Eugenio Ugo Luigi Heinzl, Michela Minero, Silvia Michela Mazzola

**Affiliations:** 1 Dipartimento di Medicina Veterinaria, Università degli Studi di Milano, via Celoria 10, 20133 Milano, Italy; francesca.dai@unimi.it (F.D.); simona.cannas@unimi.it (S.C.); michela.minero@unimi.it (M.M.); silvia.mazzola@unimi.it (S.M.M.); 2 Direzione Sicurezza, Sostenibilità e Ambiente, Università degli Studi di Milano, Via S. Sofia 9, 20122 Milano, Italy; eugenio.heinzl@unimi.it

**Keywords:** donkeys, transport, stress, welfare, chromogranin A

## Abstract

**Simple Summary:**

Transportation is recognized as a stressful animal husbandry procedure, inducing both short term and prolonged effects on welfare. Donkeys are transported for several purposes; therefore, validating a reliable and non-invasive stress indicator is pivotal. This study aimed to investigate whether salivary chromogranin A (CgA) concentration, known as an index of stress in humans and pigs, could represent a novel index of transportation-induced stress in donkeys. The research involved the measurement of salivary CgA in 19 donkeys, 15 min before and 15 min after two transportations. The transportation, which took place on two consecutive days, followed the routine procedures of the farm. The analysis of salivary CgA was carried out by an enzyme-linked immunosorbent assay test. Results showed that CgA salivary levels significantly decreased after both transportations. The physiological mechanisms underlying this result may be related to catestatin acting as an inhibitor of catecholamine release; however, due to the limited number of subjects involved, this hypothesis requires further investigation.

**Abstract:**

Road transport is known to be a stressful animal husbandry procedure as it induces the activation of two main physiological stress-related pathways: the hypothalamic-pituitary-adrenal cortex axis and the sympathetic-adrenal medulla axis. This preliminary study aimed to investigate whether salivary chromogranin A (CgA) concentration, known as a biomarker of the sympathetic activity system during psychological stress, may represent a novel physiological index of transportation-induced stress in donkeys. Nineteen Romagnolo donkeys, raised in groups on paddocks, were subject to two transportations, following the farm’s routine procedures, for a mean duration of 64 min each on two consecutive days. Salivary samples were gently collected 15 min before and 15 min after each transportation. Salivary CgA was measured by a commercially available enzyme-linked immunosorbent assay test. Results showed that CgA salivary levels significantly decreased after both transportations. The physiological mechanisms underlying this result may be related to catestatin activity, a bioactive product of the proteolytic cleavage of CgA, that acts as an inhibitor of catecholamine release. This hypothesis requires further investigation, particularly considering the limited number of subjects involved in this preliminary study. The identification of a reliable and non-invasive stress-marker would represent a useful tool for improving farm animals’ welfare in transport conditions.

## 1. Introduction

Worldwide, it is estimated that there are approximately 50 million donkeys [1]; across Europe, donkeys are kept for a variety of purposes, such as pets, leisure activities, therapy programs, or milk and meat production [2]. Therefore, they can be transported not only for reaching the slaughterhouse but also for sport activities or reaching people in need of therapy, changing owner, reaching veterinary clinics, breeding, small fair events, or sale [3]. Transportation is one of the leading husbandry practices identified as a cause of stress that may produce changes in animals’ clinical and biochemical parameters [4,5,6,7,8]. Several potential stressors are intrinsically related to transport, including loading, unloading, and confinement in unfamiliar environments; overcrowding; mixing different age groups; vibrations; changes in temperature and humidity; inadequate ventilation; noise; poor vehicle design and road conditions; duration of the journey; and often deprivation of food and water [9,10,11]. Under such circumstances, the stress response has the function of providing the energy needed in order to cope with these challenges through the activation of two main physiological pathways: the hypothalamic-pituitary-adrenal cortex axis (HPA) and the sympathetic-adrenal medulla axis (SAM). Once the HPA and SAM axes are activated, they trigger specific physiological changes that can be measured to assess the degree of activation [3]. The activities of the SAM system can be biochemically evaluated as an objective index of stress, but to obtain reliable data, it is crucial to have an accurate and non-invasive sampling method. The use of saliva rather than blood has the advantages of non-invasiveness, rapidity of sample collection, ease of collection, and the ability to maintain subject mobility during collection [12]. Catecholamines are commonly used as a sensitive biochemical index of stress-induced SAM system activation, but they are difficult to measure in saliva due to low concentration and rapid degradation [13,14]. Little research has been carried out on donkeys’ responses to transportation related stress, mainly focusing on adrenocorticotropic hormone and cortisol level variations [10,15] or free iodothyronine [8]. Recently, during an investigation of the surrogates of catecholamines that are detectable in saliva, chromogranin A (CgA) was determined to be a useful index of psychological stress [16]. CgA is an acidic glucoprotein that is co-localized with catecholamines in the secretory granules of a wide variety of endocrine structures and neurons [17]. CgA and catecholamines are co-released into an extracellular environment when the SAM system is stimulated. The adrenal medulla is the primary source of circulating CgA, while adrenergic nerve endings and neuroendocrine cells secrete CgA in peripheral tissues [16]. In humans, CgA concentration is used as an index of stressed conditions. Compared to catecholamines, CgA has greater stability in the circulatory system and therefore may be a more accurate index of sympathetic activity system during the stress response [18,19]. Detection of salivary CgA levels may have a higher analytical and diagnostic performance, as salivary sampling is non-invasive and, unlike the circulating form, CgA in the saliva is not bound to other proteins [20]. Moreover, equine CgA expression has been confirmed and described [21]. These findings led us to hypothesize that salivary CgA concentrations, known as a biomarker of sympathetic activity system, may represent a novel physiological index of transportation-induced stress in donkeys. This preliminary study aimed to investigate this hypothesis since, to the best of our knowledge, the scientific literature contains no readily-available information about this topic.

## 2. Materials and Methods 

The study included nineteen Romagnolo donkeys (M = 15; F = 4), aged 443.4 ± 148.4 days, kept for meat production on a farm situated in Northern Italy. No donkeys were transported only for data collection for the purposes of this study; all transportations were part of the farm’s breeding procedures. Since the farm was limited in size, all planned transportations were monitored. Despite this, a small number of animals were available, unbalanced by gender. In the territory considered, there were no donkey farms with similar management; therefore, for this pilot study, the number of subjects observed was not integrated with others from different breeding systems, in order to avoid data bias.

The donkeys were kept on pasture with their mothers and other donkeys of different ages. Animals were free to graze and, when needed, received hay and mixed feed; both automatic drinkers and buckets were used to provide fresh water ad libitum. All donkeys were used for human contact. All donkeys were transported twice, on two consecutive days, from January to July, using the same truck, in small groups (two to four donkeys per transportation, coming from the same familiar group). The truck’s internal dimensions (therefore the space available for the animals) were 2.50 by 6.50 m, with mobile partitions which were kept open. A spotlight illuminated the van, and the vents were kept open, compatibly with the weather conditions. In the first transportation (Trip 1), animals left the pasture to reach the farm, where they were housed in a shelter with straw bedding and provided with hay ad libitum and access to clean water. The transportation started at around 4:30 pm and its duration ranged from 50 min to 88 min (mean 64.69 ± 14.57 min). The day after, at around 4 am, donkeys were transported to the slaughterhouse (Trip 2). This transportation lasted from 60 to 75 min (mean 64.75 ± 5.54 min). Animals were always transported with conspecifics from the familiar group, with two to four donkeys per transportation. The farm manager and groom performed all the transportation procedures (loading, travel, and unloading) according to the on-farm routine. They conducted the donkeys with a lead rope, which they were accustomed to, gently encouraging them to get on and off the truck, and they also offered them food. Transportations were conducted in compliance with Council Regulation (EC) No 1/2005 of 22 December 2004 on the protection of animals during transport and related operations. Verbal informed consent was gained from the farmer prior to taking part in this research. Written consent was deemed unnecessary as no personal details of the participants were recorded. 

Saliva collection is recognized to be non-invasive, and no animal underwent more than minimal distress. Saliva samples were collected using SalivaBio Children’s Swabs (Salimetrics^®^, Carlsbad, CA, USA) 15 min before loading when donkeys were at pasture, and again at the end of the transportations, 15 min after unloading from the truck. The swab was inserted into the donkey’s mouth, while the animal was gently restrained with a head collar, without isolating it from the rest of the herd; the donkey was free to chew the swab for 1–2 min without constraint. The sampling procedure lasted no more than 5 min for each donkey, the animals being accustomed to the halter and to being handled by the groom. After the sampling, the swab was put in the device tube, which was closed with a plastic stopper to prevent evaporation and placed in ice and stored at −20 °C immediately after it arrived at the laboratory. The temperature was maintained until analysis. At the time of analysis, the samples were thawed at room temperature and centrifuged (3500 rpm for 15 min, at 4 °C) according to the protocol for salivary samples described by the kit producer. The analysis was performed using a commercially available enzyme-linked immunosorbent assay (ELISA) sandwich kit for the accurate quantitative detection of horse chromogranin A (Li StarFish Srl, Milan, Italy), validated for saliva samples. Samples were aliquoted into wells in duplicate (40 μL) and, at the end of the procedure, performed as indicated by the kit producer manual, the absorbance was measured using a wavelength of 450 nm in a microplate plate reader (Multiskan EX, LabSystem, Thermo Fisher Scientific, Milan, Italy). The average intra- and inter-assay coefficients of variation, respectively, were 6.5% and 8.3%. The assay sensitivity was 0.67 ng/mL. Mean intra-assay coefficient of variations for the final samples were 4.56%.

The laboratory researcher was blinded to the hypotheses and conditions.

Data were entered into Microsoft Excel (Microsoft Corporation, 2010, Washington, DC, USA) before being analyzed with the SPSS statistical package (SPSS Statistic 25, IBM, Armonk, NY, USA). A descriptive analysis was first performed to determine the mean and standard deviation of CgA concentration. A match-paired Wilcoxon’s test was used to compare concentrations from pre- to post-transportation in both transportations (Trip 1 and Trip 2) and pre- and post-transportation CgA concentration between transportations. *p*-values ≤ 0.05 were deemed statistically significant.

## 3. Results

**Figure 1:**
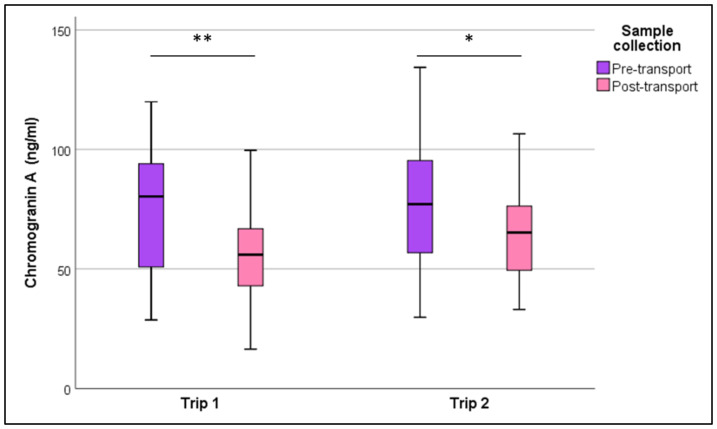
Chromogranin A (ng/mL) pre- and post-transportation drawn in a box plot for both trips; lines extending from the whiskers indicate the variability outside the upper and lower quartiles. (match-paired Wilcoxon’s test ** *p* < 0.01; * *p* < 0.05).

Our results showed that, in donkeys, chromogranin A levels significantly decreased after transportation (match-paired Wilcoxon’s test, *p* < 0.05) (Figure 1). CgA values during the first transportation (both pre- and post-transportation) were not significantly different from those of the second transportation (match-paired Wilcoxon’s test, *p* > 0.05). 

## 4. Discussion

To the authors’ knowledge, chromogranin A salivary concentration has not been measured before in donkeys. The primary structure of the equine nucleotide sequence encoding CgA was determined by Sato et al. [22], which also compared the aminoacidic sequence of equine CgA with those of human, porcine, bovine and other species, showing high conservation of the NH2 terminal-1-177 and COOH terminal 314–430 aminoacidic regions. This result made us hypothesize that the commercially available CgA ELISA kit for horses, tested for the accurate quantitative detection of chromogranin A in serum, plasma, urine, saliva, and tissue homogenates, had adequate sensitivity for CgA titration in donkeys. In horses, road transportation is known to activate the hypothalamic-pituitary-adrenal cortex and the sympathetic and adrenal medullary systems [5,6,10,20,21,23], but, to the best of our knowledge, the change in CgA has not yet been tested. Differently from what could be expected, in our study, the salivary concentration of CgA decreased after each of the two considered transportations. Although, in the present study, all possible measures were taken to minimize stressors (e.g., not mixing the animals with unknown conspecifics, using the same truck, transporting them for short distances, gentle handling by known stockman), at the end of the transports, we expected an increase in salivary CgA concentration as a reflection of sympathetic-adrenal medullary axis activation. Our results could be partially related to what was observed by Fazio et al. [24], who investigated the effects of different human handling on young horses in stabled conditions and during the short-transport road. They demonstrated that the quality of handling to which horses were subjected could affect the stress response produced by transportation. The role of salivary CgA as a marker of stress is described in the literature in pigs [25], dogs [13], and calves, with results not always consistent. Escribano et al. [25] described the validation of a time-resolved immunofluorometric CgA assay and its application as a marker of acute stress in porcine saliva in a model of acute experimental stress, in which animals were immobilized for 3 min with a nose snare. The authors evidenced a significant increase in salivary CgA levels at 15 min post-stressor stimulus. Since the time course of the changes of CgA salivary levels post-stress is not yet known, in the present study, we opted to utilize the timing suggested by Escribano [25]. Ott et al. [26] investigated salivary CgA as a biomarker for pigs’ stress evaluation, and their results indicated that salivary CgA reacts differently to different types of stressors. They exposed pigs to stressors that cause significant social and physiological changes: the mixing of unfamiliar animals (a procedure associated with the increase in stress metabolites, the suppression of the immune function, growth retardation, and skin lesions), and fasting and refeeding. The mixing of unfamiliar pigs induced a decrease in CgA salivary concentrations which conversely showed a significant elevation after the feed deprivation period [26]. 

In Escribano et al. [27], the CgA levels in pigs’ saliva showed an increase both during transportation and housing in the slaughterhouse and after applying a psychosocial stress model, where finishing pigs were mixed with familiar animals after a previous period of isolation. More recently, Escribano et al. [28] evaluated salivary CgA concentration at weaning, known to induce acute stress in pigs. They observed a highly significant increase in CgA in saliva one day after weaning, compared to the pre-weaning levels. The authors hypothesized that weaners, subjected to mother–young detachment, undergo neuroendocrine and behavioral consequences and are subject to other stressors like uncertainty, social hierarchy re-establishment, and fear of the novel environment. These stimuli have demonstrated the induction of the “flight and fight” response in pigs which is associated with the activation of the SAM system [28]. In a 2010 study, Ogino et al. investigated the influence of social isolation and transportation on CgA salivary levels in calves. They evidenced that social isolation induced a significant decrease in the salivary CgA concentration, while transport evoked a rise, even if not statistically significant. 

In humans, CgA is considered a sensitive and reliable index of psychological stress. Nakane et al. [19] showed that salivary CgA levels were elevated before subjects gave an oral presentation and then decreased immediately after. Fujimoto et al. [29] measured salivary CgA levels in two groups of people (young and elderly) in order to evaluate mental stress during upper gastrointestinal endoscopies. They compared CgA salivary levels at rest, before endoscopy, and during endoscopy and highlighted that, in both groups, CgA levels decreased significantly during endoscopy, more so in the elderly patients. 

Our results in donkeys seem consistent with what has been shown in the study by Yamakoshi et al. [30]. They evaluated salivary CgA in a stressful situation created by simulated monotonous driving and evidenced that CgA levels fell gradually in accordance with the gradual increase of the subjective rating of stress and significantly decreased over the two hours of simulated monotonous driving, in which the driver felt considerably stressed [30].

A possible hypothesis explaining why CgA levels can be affected differently according to different stressors and exposure times is related to catestatin action [31]. Catestatin is a bioactive product of the proteolytic cleavage of CgA that acts as a potent inhibitor of catecholamine (CA) nicotinic cholinergic stimulated secretion, desensitization of catecholamine release. This peptide exhibits potent catecholamine release-inhibitory activity by acting on the neuronal nicotinic acetylcholine receptors and transcription of chromogranin A gene [32,33]. CgA and CA are co-released into the extracellular environment, and CA mediates sympathoadrenal activity on cardiovascular target cells in order to increase blood pressure. Yamakoshi et al.’s findings demonstrated that sympathetic-induced vasomotor constriction was stimulated during the two hours of monotonous driving simulation: peripheral vessel constriction induced a significative increase in total peripheral resistance, a significative decrease of normalized pulse volume, and an increase in blood pressure, which are associated with an increase in the subjective rating of stress [30]. 

The stimulus analyzed in Yamakoshi’s research could probably be compared to the 50–80 min journey, without particularly acute stressful conditions, to which the donkeys of the present study were subjected. Yamakoshi’s results indicate that the two hours of monotonous driving evoked considerable stress in drivers, resulting in a gradual rise in blood pressure caused by an increase in sympathetic-induced vasoconstriction, rather than by sympathoadrenal activity, that is not activated in the monotonous situation [30]. It is known that the increase in blood pressure is correlated with catestatin activation and that catestatin is associated with augmented baroreflex sensitivity [33,34]. Moreover, catestatin acts as negative feedback on CgA secretion, which is blocked by this peptide fragment [32]. This mechanism could explain why CgA levels decreased during the simulated monotonous driving in Yamakoshi’s research, although CgA is a feasible marker of stress. We hypothesize that this physiological mechanism could be the basis of the decrease in the salivary concentration of CgA observed after transportation in the donkeys. If confirmed in future studies, this hypothesis would further prove what has been recently found by Srithunyarat et al. [35] in a study on dogs, when they reported that canine psychological stress was associated with an increased level of salivary catestatin.

## 5. Conclusions

Since this study involved only a limited number of donkeys, additional research considering a larger animal sample is needed to confirm ELISA’s reliability for donkeys’ salivary CgA levels and establish its physiological range in resting conditions. Measuring salivary CgA concentrations in donkeys may represent a promising tool for obtaining information about their stress response through a non-invasive technique that is not influenced by sampling and can be easily carried out on many animals directly in on-farm conditions.
